# Supplementary material for: Therapeutic Potential of Peucedanum japonicum Thunb. and Its Active Components in a Delayed Corneal Wound Healing Model Following Blue Light Irradiation-Induced Oxidative Stress
Source: Antioxidants (Basel). 2023 May 29;12(6):1171. doi: 10.3390/antiox12061171 (PMC10295055; doi:10.3390/antiox12061171)
Supplement: Supplementary file 1 [file antioxidants-12-01171-s001.zip › antioxidants-2395807-supplementary.pdf]

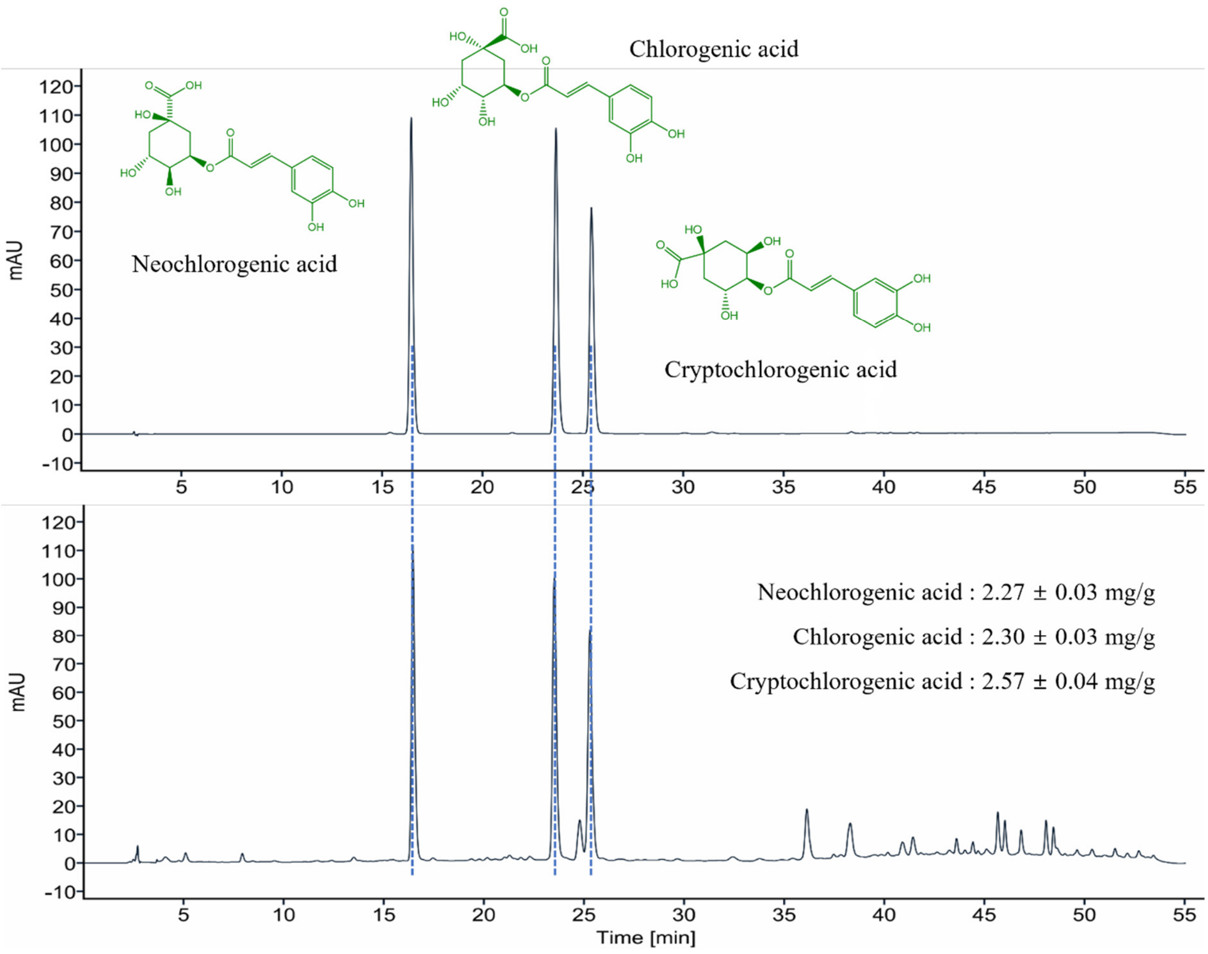


**Figure S1. HPLC chromatograms of chlorogenic acid (CA), neochlorogenic acid (NCA), and cryptochlorogenic acid (CCA) standards compound and *Peucedanum japonicum* Thunberg leaf extract (PJE).**

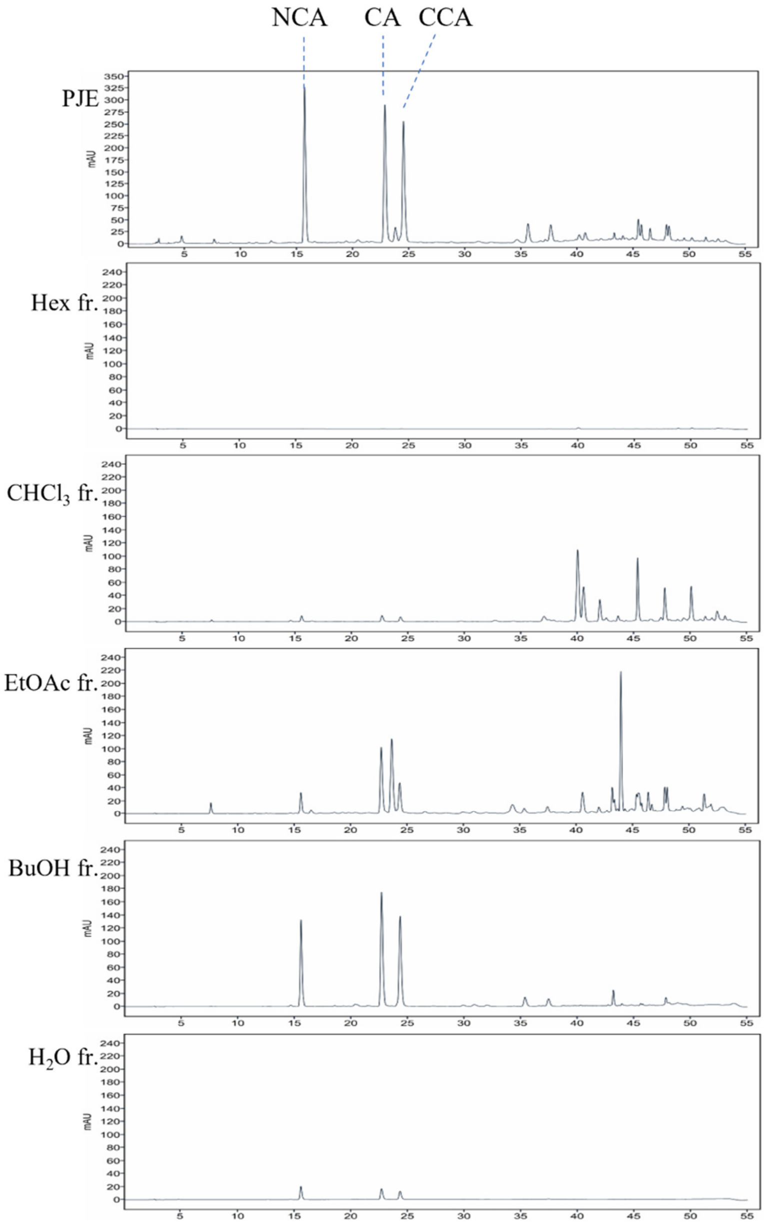


**Figure S2. HPLC chromatograms of PJE and its fractions after solvent fractionation.** The CA, NCA and CCA peaks are indicated.

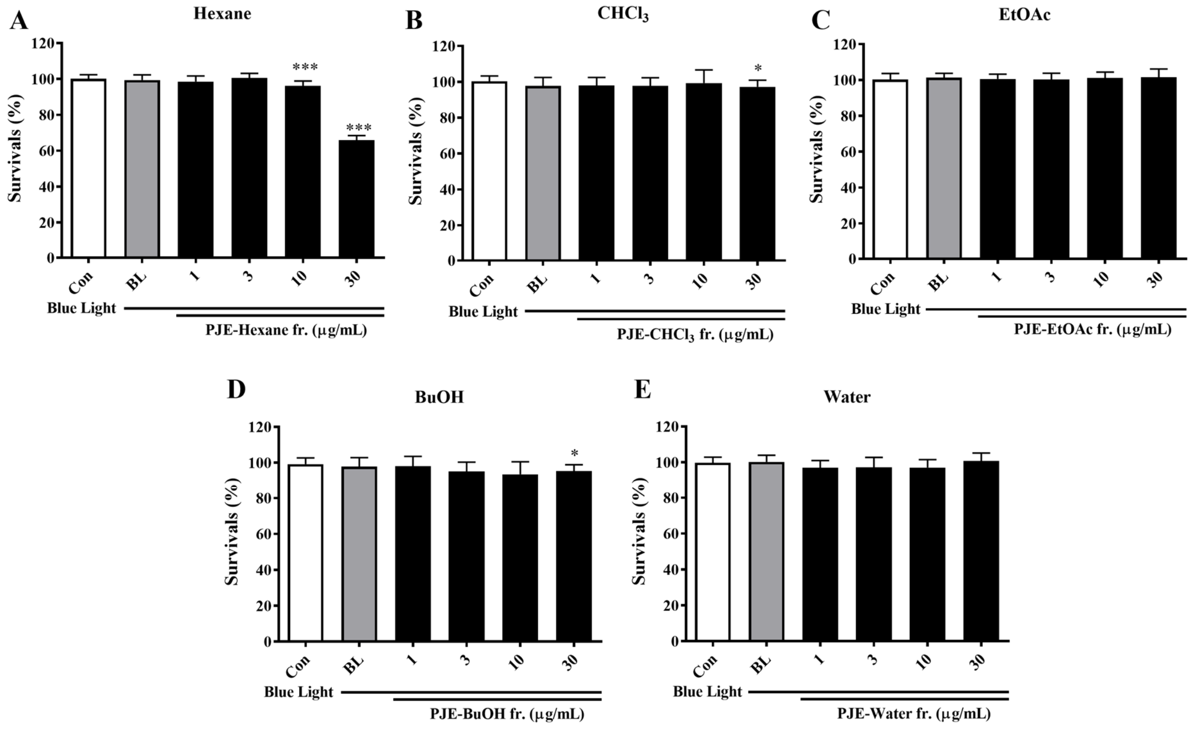


**Figure S3. Effects of PJE solvent fractionation on HCEC survival.** The data are presented as the means ± SDs. $^{*}P<0.05$, $^{**}P<0.01$, $^{***}P<0.001$ compared to BL.

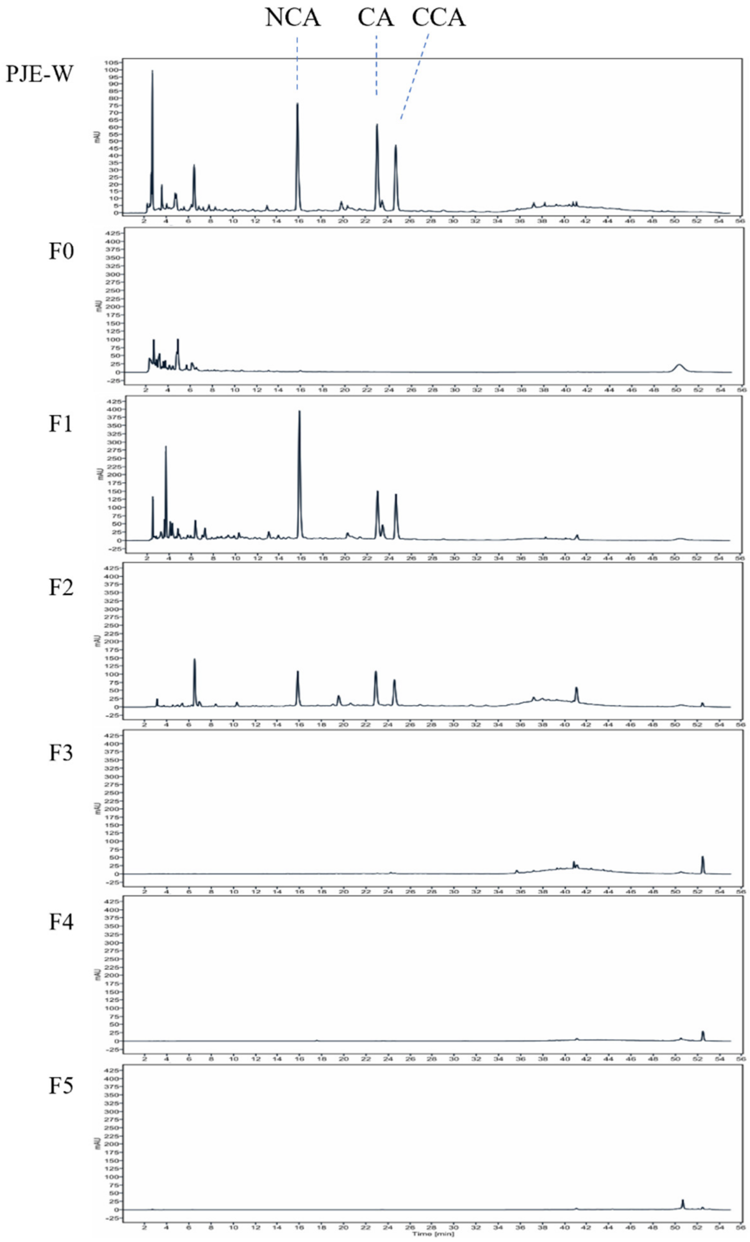


**Figure S4. HP-20 open column chromatography of PJE/W and its fractions.** The CA, NCA and CCA peaks are indicated.

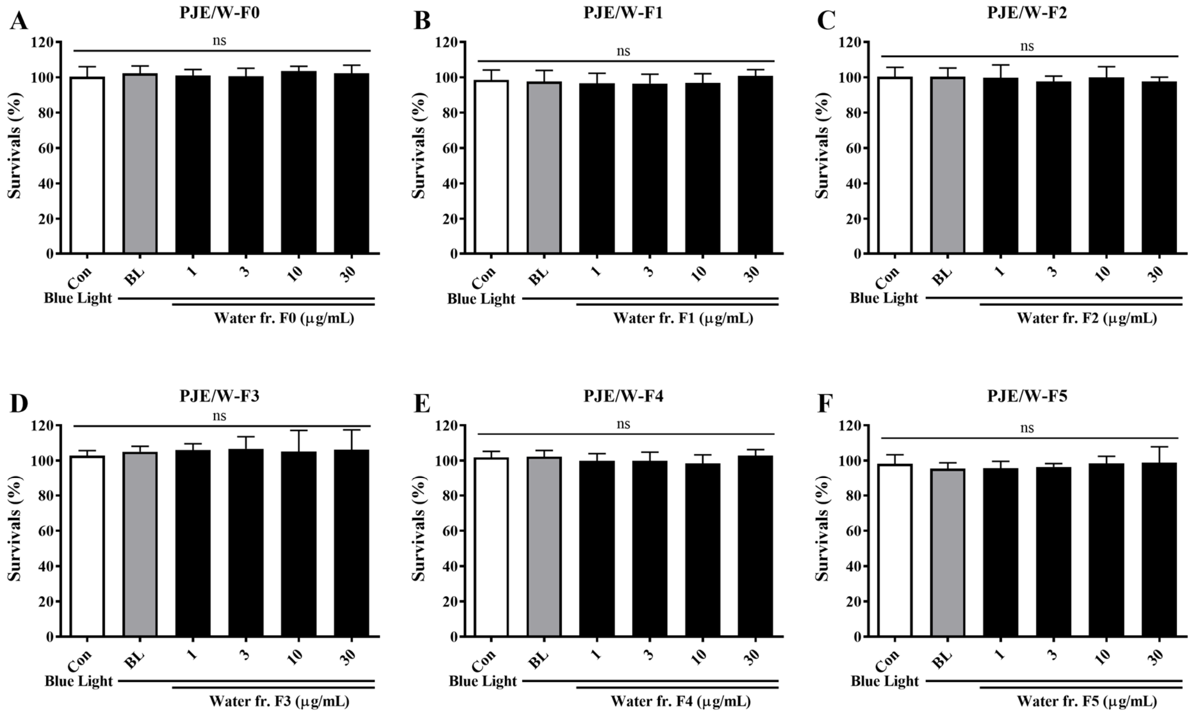


**Figure S5. Effects of HP-20 open column chromatography fractionation of PJE/W on HCEC survival.** The data are presented as the means ± SDs. ns, not significant.

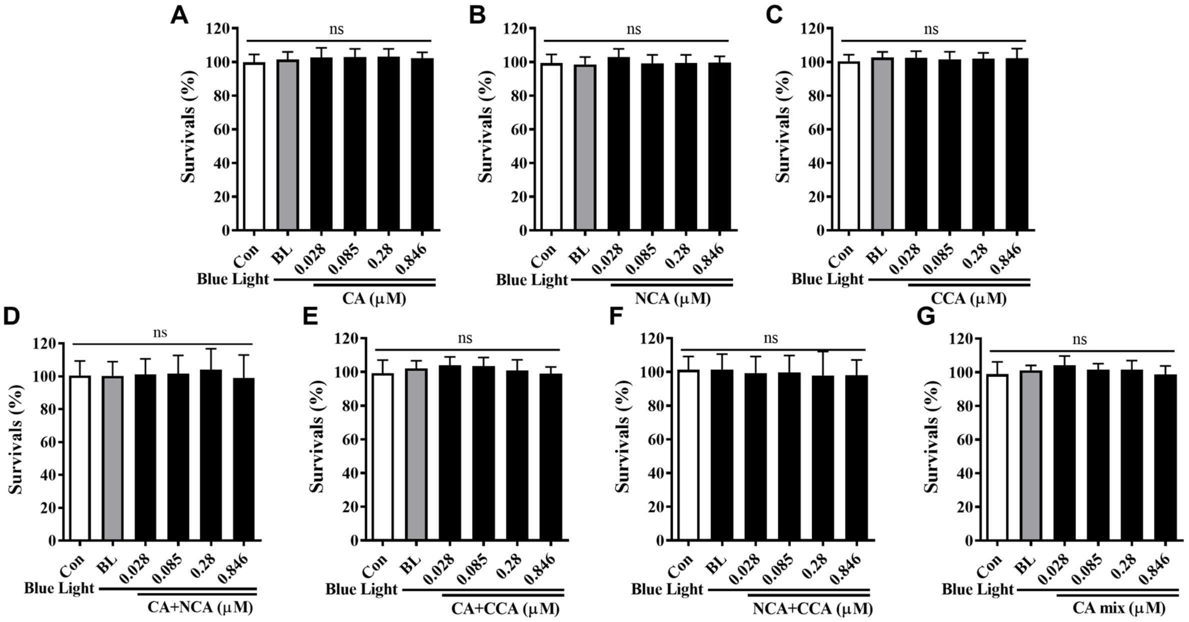


**Figure S6. Effects of the major compounds of PJE on HCEC survival.** The data are presented as the means ± SDs. ns, not significant.
